# In Vitro Susceptibility of *Candida* Species to Four Antifungal Agents Assessed by the Reference Broth Microdilution Method

**DOI:** 10.1155/2013/236903

**Published:** 2013-10-22

**Authors:** Fahriye Eksi, Efgan Dogan Gayyurhan, Iclal Balci

**Affiliations:** Department of Medical Microbiology, Faculty of Medicine, Gaziantep University and Universite Bulvari, 27310 Gaziantep, Turkey

## Abstract

This study was performed to determine the distribution of *Candida* species isolated from the blood cultures of the patients hospitalized in our hospital and to investigate their antifungal susceptibility. *Candida* strains were identified at species level by using classical methods and API ID 32C (bioMerieux, France) identification kits. The susceptibility of the strains to amphotericin B, fluconazole, voriconazole, and caspofungin was evaluated by using the reference broth microdilution method in document M27-A3 of the Clinical and Laboratory Standards Institute. Of the 111 *Candida* strains isolated, 47.7% were identified as *C. albicans* and 52.3% as non-albicans *Candida* strains. The MIC ranges were 0.03–1 **μ**g/mL for amphotericin B, 0.125–≥64 **μ**g/mL for fluconazole, 0.03–16 **μ**g/mL for voriconazole, and 0.015–0.25 **μ**g/mL for caspofungin. All *Candida* strains were susceptible to amphotericin B and caspofungin. 10.8% isolates were resistant to fluconazole and 8.1% isolates were dose-dependent susceptible. While 0.9% isolate was resistant to voriconazole, 0.9% isolate was dose-dependent susceptible. In our study, *C. albicans* and *C. parapsilosis* were the most frequently encountered agents of candidemia and it was detected that voriconazole with a low resistance rate might also be used with confidence in the treatment of infections occurring with these agents, primarily besides amphotericin B and caspofungin.

## 1. Introduction

The species of fungi included in genus *Candida* are normally microorganisms which are found in the flora of the human skin and mucosa. Oral colonization by *Candida albicans* has been reported at 17.7% in the healthy population. Among hospitalized patients, oral carriage of *Candida albicans* rises to 40.6%. The infections which occur with pathogenic fungi, *Candida* species in particular, are both common and increasing in frequency [1]. Candidemia and invasive candidiasis are a major cause of nosocomial infections, linked to a number of risk factors such as prior antimicrobial therapy, venous and urinary catheters, intensive care unit admission, parenteral nutrition, major surgery, and immunosuppressive therapies [2, 3]. Although *C. albicans* is the most frequent agents of candidemia, it has shown an increasing rate of nonalbicans *Candida* species over the past decades. Compared with incidences from the 1980s, a larger proportion of *Candida* bloodstream infections are now caused by *Candida glabrata* in the United States and by *Candida parapsilosis* and *Candida tropicalis* in European, Canadian, and Latin American hospitals. The most important reasons for the increase in candidemia depending on nonalbicans *Candida* strains are the prophylactic and empirical administration of antifungals, particularly of azole drugs due to their easy use and to their large spectrum [4]. In many studies, both the incidence and agent spectrum of candidemia agents were reported [5].

Even though the number of systemically effective antifungal agents used in the treatment of *Candida* infections is not very large, several antifungals may be used including amphotericin B, azole group of drugs, flucytosine, and echinocandins [6]. The choice of initial antifungal agent for the empirical treatment of a suspected invasive *Candida* infection is also difficult. Development of resistance to the antifungal agents used in *Candida* infections is a serious problem. The use of fluconazole prophylaxis over the last two decades is believed to have led to an increase in nonalbicans species with reduced fluconazole susceptibility [7]. Therefore, there is an increasing need for in vitro antifungal susceptibility testing to choose the most appropriate and effective antifungal agent. For this purpose, the Clinical and Laboratory Standards Institute (CLSI) issued reports in order to identify the standard methods in both yeasts and molds. Of them, M27-A3 Reference Method for Broth Dilution Antifungal Susceptibility Testing of Yeasts, Approved Standard, is the microdilution method developed to determine the susceptibility of yeasts to antifungal agents [8].

This study aimed to identify the *Candida* species isolated from the blood cultures of the patients hospitalized in various clinics, particularly the intensive care clinic of our hospital, at species level and to determine their susceptibility to amphotericin B, fluconazole, voriconazole, and caspofungin by using the standard broth microdilution method.

## 2. Materials and Methods

This study was approved by the Ethics Committee of the Faculty of Medicine at Gaziantep University. The 111 *Candida* isolates collected from blood cultures from patients hospitalized in various clinics of the Medical Faculty Hospital at Gaziantep University between March 2008 and January 2009 were evaluated. Of the blood cultures, 39 (35.1%) were obtained from patients hospitalized in the intensive care clinic, 15 (13.5%) in the podiatry clinic, 15 (13.5%) in the pediatric oncology clinic, 9 (8.1%) in the internal medicine clinic, and the remaining 33 (29.8%) in the other clinics. The germ tube test, microscopic appearance on Cornmeal Tween 80 agar, determination of the colour of colonies on Candida ID 2 agar—a chromogenic medium—and API ID 32C (bioMerieux, France) identification kit were used for the identification of yeast strains.

Antifungal susceptibility testing was performed using the broth microdilution method. The Clinical and Laboratory Standards Institute developed and published an approved reference method for the broth microdilution testing (CLSI document M27-A3) of *Candida* species [8]. The standard powders of fluconazole (Sigma), amphotericin B (Sigma), voriconazole (Pfizer), and caspofungin (Merck) were used as antifungals. Distilled water was used as a solvent for fluconazole and caspofungin, whereas DMSO (dimethylsulphoxide) (Sigma) was used as a solvent for water-insoluble amphotericin B and voriconazole. The stock solutions were prepared at the rate of 1280 *μ*g/mL for fluconazole, 1600 *μ*g/mL for amphotericin B, 1600 *μ*g/mL for voriconazole, and 1600 *μ*g/mL for caspofungin. For the susceptibility test, RPMI 1640 (with glutamine, bicarbonate-free, and containing phenol red as the pH indicator) (Sigma) was used as a medium. The final concentrations were in the range 64–0.125 *μ*g/mL for fluconazole, 16–0.03 *μ*g/mL for amphotericin B and voriconazole, and 8–0.015 *μ*g/mL for caspofungin. Each *Candida* strain was studied twice for fluconazole, amphotericin B, voriconazole, and caspofungin. The results were evaluated 24 hours later for caspofungin and 48 hours later for fluconazole, amphotericin B, and voriconazole. For amphotericin B, the MIC endpoint was defined as the lowest drug concentration that resulted in a reduction in growth by 90% or more, compared with that of a drug-free growth control well. For fluconazole, the MIC endpoint was defined as a 50% reduction in optical density. For caspofungin, the endpoint was defined according to the recently published reports that analyzed influences of methodological variables on susceptibility testing of caspofungin against *Candida* species, though an interpretive cut-off value is not yet available for this echinocandin [9]. Therefore, the endpoint is given as the concentration of the drug in the assay at which 50% of growth control was observed. The in vitro susceptibility tests of the isolated *Candida* species were interpreted considering the guide prepared by the CLSI [10]. Although no resistance extreme was specified for amphotericin B by the CLSI, the strains with a MIC value of >1 are accepted as resistant [8]. Since *C. krusei* is an intrinsically fluconazole-resistant *Candida* species, no resistance extremes were used in the interpretation of the MIC values obtained for *C. krusei* [10]. Quality control was ensured by testing the CLSI-recommended strains *C. parapsilosis* ATCC 22019 and *C. krusei* ATCC 6258 [10].

Statistical significance was determined by chi-square analysis (Epi Info, version 6).

## 3. Results

Of the 111 *Candida* strains isolated, 53 (47.7%) were identified as *C. albicans*, 41 (36.9%) as *C. parapsilosis*, 6 (5.4%) as *C. krusei,* 3 (2.7%) as *C. tropicalis*, 3 (2.7%) as *C. glabrata,* 2 (1.8%) as *C. kefyr,* and 2 (1.8%) as *C. lusitaniae*, while one (0.9%) was identified as *C. famata* (Table 1). The MIC ranges and MIC_50_ and MIC_90_ values of the identified strains according to the antifungal agent are given in Table 2.

All *Candida* strains isolated were detected to be susceptible to amphotericin B and caspofungin.

Fluconazole resistance and dose-dependent susceptibility were detected in 3 (5.66%) and 6 (11.3%) of the *C. albicans* isolates (*n* = 53), respectively, and no resistance was detected for voriconazole.

Of the nonalbicans *Candida* strains (*n* = 58), 9 (15.5%) were found resistant to fluconazole (all *C. krusei* strains were accepted as resistant), while 3 (5.2%) were found dose-dependent susceptible. Of the 6 *C. krusei* strains isolated, 4 strains were found to have a MIC value of ≥64 *μ*g/mL and 2 strains were found to have a MIC value of 16 *μ*g/mL. Since the *C. krusei* strains were intrinsically resistant to fluconazole, all of them were accepted as resistant to fluconazole regardless of the MIC values determined in vitro. Of the 3 strains that were resistant to fluconazole except for *C. krusei*, one was detected as *C. tropicalis* and 2 were detected as *C. glabrata*. Of the 3 strains detected to be dose-dependent susceptible, one was detected as *C. glabrata* (MIC = 16 *μ*g/mL), one as *C. famata* (MIC = 16 *μ*g/mL), and one as *C. parapsilosis* (MIC = 16 *μ*g/mL). No statistically significant difference in fluconazole resistance was found between *C. albicans* and nonalbicans strains (*χ* ^2^ = 2.79, *P* = 0.094).

Voriconazole resistance was detected in one (1.72%) of the nonalbicans *Candida* strains, while dose-dependent susceptibility was detected in one (1.72%) of them. *C. tropicalis* was detected as the strain that was resistant to voriconazole and *C. glabrata* as the dose-dependent susceptible strain. No statistically significant difference in voriconazole resistance was found between *C. albicans* and nonalbicans strains (*χ* ^2^ = 0.92, *P* = 0.336).

## 4. Discussion

The frequency of invasive mycoses due to opportunistic fungal pathogens has increased significantly over the past two decades. More than 17 different species of *Candida* have been identified as etiologic agents of bloodstream infections. Approximately 95% of all *Candida* bloodstream infections are caused by four species: *C. albicans*, *C. glabrata*, *C. parapsilosis*, and *C. tropicalis* [11]. Of the *Candida* strains isolated in our study, 47.7% were identified as *C. albicans*, 36.9% as *C. parapsilosis*, 5.4% as *C. krusei,* 2.7% as *C. tropicalis*, 2.7% as *C. glabrata*, and the remaining 4.5% as *C. kefyr*, *C. famata*, and *C. lusitaniae*. These results are compatible with the other research results [12, 13]. *C. albicans* almost always ranks first, while the frequency of nonalbicans species varies according to various studies. In many studies performed in the recent years, it has been expressed that there has been an evident change in agents of candidemia and that the rate of candidemia depending on nonalbicans *Candida* strains has reached approximately 50% [14]. Although *C. albicans* was the most frequently observed species in the study that was carried out by Malani et al. [12] and that investigated agents of candidemia in a 12-year period between 1988 and 1999, its proportion decreased from 63% to 43%. Nevertheless, the incidence of *C. glabrata* increased to 20% from 10%, while the incidence of *C. parapsilosis* increased to 18% from 5%. Cuenca-Estrella et al. [13] identified some 351 *Candida* strains isolated from the blood samples and reported that they had found *C. albicans* at the rate of 51%, *C. parapsilosis* at the rate of 23%, *C. tropicalis* at the rate of 10%, *C. glabrata* at the rate of 9%, and *C. krusei* at the rate of 4%. In 2003, Messer et al. [15] examined some 1,397 *Candida* strains, most of which were isolated from the blood culture, from North America, Europe, and Latin America within the scope of SENTRY Antimicrobial Surveillance Program and they identified 48.7% of them as *C. albicans*, 17.3% of them as *C. parapsilosis*, 17.2% of them as *C. glabrata*, 10.9% of them as *C. tropicalis*, 1.9% of them as *C. krusei*, and 4% of them as other *Candida* species. *C. parapsilosis* is notorious for the ability to form biofilms on catheters and other implanted devices, for nosocomial spread by hand carriage, and for persistence in the hospital environment. It is also well known for causing infections in infants and neonates [3].

The increase in fungal infections has prompted an increase in the use of antifungal agents, and in practice the widespread clinical use of these agents has resulted in measurable rates of acquired or innate fungal resistance in *Candida* species [3]. A new period initiated in the treatment of fungal infections with the discovery of amphotericin B in 1953. Even though amphotericin B is one of the most toxic antimicrobial agents in clinical use, it still qualifies as a standard treatment. Resistance is reported in nonalbicans *Candida* species, for example, species *C. lusitaniae* and *C. guilliermondii* [16]. Clinical failure to respond to amphotericin B treatment occurs with the nonrecovery of the factors about the immune system of the host rather than with in vitro resistance [17]. Amphotericin B resistance may vary by region, and the resistance profile may also vary at different times in the same region. In our study, the MIC values of the 111 *Candida* strains under examination were in the range 0.03–1 *μ*g/mL for amphotericin B, and no resistant (≥2 *μ*g/mL) strain was encountered. Barchiesi et al. [18] from Italy detected that the MIC values for amphotericin B were in the range 0.03–0.5 *μ*g/mL in 56 *Candida* strains; Godoy et al. [19] from Latin America detected that the MIC values for amphotericin B were in the range 0.125–1 *μ*g/mL in 103 *Candida* strains; and Cuenca-Estrella et al. [13] from Spain detected that the MIC values of 351 *C. albicans* strains for amphotericin B were in the range 0.03–0.5 *μ*g/mL, and they did not find any amphotericin B resistance, which is parallel with our study. In various studies carried out in our country [20, 21], they did not detect any amphotericin B resistance in the *Candida* strains isolated from the blood cultures either. However, in their study, Kiraz et al. [22] found the MIC range as 0.03–4 *μ*g/mL and the MIC_50_ and MIC_90_ values as 0.5 and 1 *μ*g/mL in 300 *C. albicans* strains, respectively. On the other hand, different results were obtained in the studies performed in various countries. Diekema et al. [23] detected that the MIC values of amphotericin B in 254 *Candida* strains were in the range 0.25–2 *μ*g/mL, while they observed the MIC value as 2 *μ*g/mL in only 7 strains and reported 3% resistance. Cuenca-Estrella et al. [24] determined the MIC range of amphotericin B as 0.03–2 *μ*g/mL and the rate of resistance as 2.7% in 514 *Candida* strains obtained from Spain, whereas they determined the MIC range as 0.06–8 *μ*g/mL and the rate of resistance as 0.86% in some 230 *Candida* strains obtained from Argentina.

Today the most frequently used antifungals systemically and locally are the azole group of agents. Of the azoles used systemically, fluconazole is the most frequently used one in yeasts. Among *Candida* species, *C. krusei* is intrinsically resistant to fluconazole. Furthermore, the susceptibility of *C. glabrata* strains also varies widely. While some *C. glabrata* strains are dose dependent and fluconazole susceptible, about 15% of them display real resistance. Following prolonged fluconazole prophylaxis in patients with AIDS in particular, acquired resistance to fluconazole might develop even in *C. albicans* isolates [17]. In our study, the MIC values for fluconazole were detected between 0.125 and 64 *μ*g/mL for the 111 *Candida* strains under examination and fluconazole resistance was 5.66% in *C. albicans* strains and 15.5% in non*albicans* candida strains. The number of dose-dependent susceptible (16–32 *μ*g/mL) isolates was 9 (8.1%), and all *C. krusei* strains were accepted as resistant. Sabatelli et al. [25] detected 6.4% resistance to fluconazole in 6,595 *Candida* isolates. Skrodeniene et al. [26] found 14 (15.1%) of 93 *C. albicans* strains resistant to fluconazole, while Sojakova et al. [27] reported fluconazole resistance as 13% in 227 *Candida* isolates. The rates of resistance are reported in a quite wide range in the studies carried out in our country as well. While no resistance was seen at all in some studies [21, 22], Kantarcioglu and Yücel [28] detected 38.8% resistance in nonalbicans *Candida* strains, while Kaya et al. [29] detected 68.7% resistance in 32 *C. albicans* strains isolated from neutropenic patients with malignity and 63.2% resistance in 106 nonalbicans strains.

 Voriconazole is a synthetic triazole derived from fluconazole. As a result of structural changes, its activity of inhibiting the target enzyme lanosterol demethylase increased and its spectrum extended [30]. It is also effective on fluconazole-resistant *Candida* strains. Nevertheless, a significant number of fluconazole-resistant *Candida* isolates also become resistant to voriconazole as a result of cross-resistance besides being resistant to ketoconazole and itraconazole [17]. In our study, the MIC range of voriconazole was 0.03–16 *μ*g/mL for the 111 *Candida* strains evaluated. The number of voriconazole-resistant (≥4 *μ*g/mL) *Candida* strains was 1 (1.72%), whereas one strain (1.72%) was dose-dependent susceptible (2 *μ*g/mL). The voriconazole-resistant strain was detected as *C. tropicalis.* Accordingly, no voriconazole resistance was detected for *C. albicans* strains, while voriconazole resistance was 1.72% in nonalbicans strains. In the study where Espinel-Ingroff et al. [31] investigated the voriconazole resistance of 90 *Candida* strains, they did not detect any resistance in 20 *C. albicans* strains, whereas they detected resistance in 3 (4.3%) of 70 nonalbicans isolates. In addition, although the fluconazole resistance of *C. albicans* strains was 11/20 (55%) in this study, no voriconazole resistance was observed [31]. Swinne et al. [16] detected the MIC range of voriconazole as ≤0.008–16 *μ*g/mL in 121 nonalbicans *Candida* strains and reported resistance in 4 of them (3.3%), whereas Alexander et al. [32] detected the MIC values for voriconazole as <0.03–>64 for 212 *Candida* strains and found voriconazole resistance in 7 of them (3.3%). Likewise, Sabatelli et al. [25] found the voriconazole resistance of 6,595 *Candida* isolates as 3.3%. The results of each of these last three studies are parallel with our study. The rates of resistance quite vary in the studies carried out in our country. Keçeli Özcan et al. [33] detected voriconazole resistance in 2 (4.7%) of 43 nonalbicans *Candida* strains, whereas Aydin et al. [34] did not detect any voriconazole resistance in 166 *Candida* strains.

 Echinocandins are in general active against various *Candida* and Aspergillus spp. Their favorable activity against azole and amphotericin B resistant as well as azole and amphotericin B susceptible *Candida* strains is one of the major advantages of echinocandin use in clinical practice [17, 30]. Caspofungin and anidulafungin have been approved by the US Food and Drug Administration for the treatment of invasive candidiasis, including candidemia [3]. All of the strains examined in our study were found susceptible to caspofungin (MIC ≤ 2 *μ*g/mL). In their study, Pfaller et al. [35] did not encounter any caspofungin-resistant strains in 8,197 *Candida* isolates. Likewise, Alexander et al. [32] detected the MIC range of caspofungin as <0.03–2 *μ*g/mL in 212 *Candida* strains and they found no caspofungin resistance in any of them.

## 5. Conclusions

Our results also confirm the elevated incidence of bloodstream infections caused by nonalbicans *Candida* strains. It was concluded that voriconazole with a low resistance rate might also be used with confidence in the treatment of infections occurring with *Candida* species, primarily besides amphotericin B and caspofungin. Moreover, identification at species level in *Candida* strains is necessary particularly in terms of detecting *C. krusei* and the other species that might be resistant to fluconazole.

## Figures and Tables

**Table 1:** Distribution of *Candida* species isolated from blood cultures.

Species	*N*	%
*C. albicans *	53	47.8
*C. parapsilosis *	41	36.9
*C. krusei *	6	5.4
*C. tropicalis *	3	2.7
*C. glabrata *	3	2.7
*C. kefyr *	2	1.8
*C. lusitaniae *	2	1.8
*C. famata *	1	0.9

Total	111	100

**Table 2:** MIC ranges as well as MIC_50_ and MIC_90_ values of the identified strains according to the antifungal agent.

Species (*n*)	Antifungal agent	MIC range (*μ*g/mL)	MIC_50_ (*μ*g/mL)	MIC_90_ (*μ*g/mL)
*C. albicans *(53)	Fluconazole	0.25–32	0.5	16
	Amphotericin B	0.03–0.25	0.06	0.125
	Voriconazole	0.03–0.25	0.03	0.03
	Caspofungin	0.015–0.25	0.03	0.06

*C. parapsilosis *(41)	Fluconazole	0.25–64	0.5	1
	Amphotericin B	0.03–0.25	0.06	0.125
	Voriconazole	0.03–0.06	0.03	0.03
	Caspofungin	0.06–0.25	0.125	0.25

*C. tropicalis *(3)	Fluconazole	0.25–64	1	64
	Amphotericin B	0.125–0.25	0.125	0.25
	Voriconazole	0.03–16	0.06	16
	Caspofungin	0.015–0.06	0.06	0.06

*C. krusei *(6)	Fluconazole	16–>64	64	>64
	Amphotericin B	0.25–1	0.25	1
	Voriconazole	0.06–0.5	0.125	0.5
	Caspofungin	0.015–0.25	0.015	0.25

*C. kefyr *(2)	Fluconazole	0.25–0.5	0.25	0.5
	Amphotericin B	0.125–0.5	0.125	0.5
	Voriconazole	0.03–0.03	0.03	0.03
	Caspofungin	0.03–0.06	0.03	0.06

*C. glabrata *(3)	Fluconazole	16–>64	64	>64
	Amphotericin B	0.125–0.125	0.125	0.125
	Voriconazole	0.25–2	1	2
	Caspofungin	0.015–0.06	0.03	0.06

*C. lusitaniae *(2)	Fluconazole	0.5–2	0.5	2
	Amphotericin B	0.125–0.125	0.125	0.125
	Voriconazole	0.03	0.03	0.03
	Caspofungin	0.06–0.06	0.06	0.06

*C. famata *(1)	Fluconazole	16	16	16
	Amphotericin B	0.25	0.25	0.25
	Voriconazole	0.25	0.25	0.25
	Caspofungin	0.03	0.03	0.03
